# Genome-Wide Association Study and Gene Specific Markers Identified 51 Genes or QTL for Resistance to Stripe Rust in U.S. Winter Wheat Cultivars and Breeding Lines

**DOI:** 10.3389/fpls.2020.00998

**Published:** 2020-07-03

**Authors:** Jingmei Mu, Lu Liu, Yan Liu, Meinan Wang, Deven R. See, Dejun Han, Xianming Chen

**Affiliations:** ^1^ Department of Plant Pathology, Washington State University, Pullman, WA, United States; ^2^ College of Life Science and Food Engineering, Hebei University of Engineering, Handan, China; ^3^ State Key Laboratory of Crop Stress Biology for Arid Areas and College of Agronomy, Northwest A&F University, Yangling, China; ^4^ Wheat Health, Genetics and Quality Research Unit, Agricultural Research Service, U.S. Department of Agriculture, Pullman, WA, United States

**Keywords:** stripe rust, winter wheat, resistance, genome-wide association study, molecular markers, yellow rust

## Abstract

Stripe (yellow) rust, caused by fungal pathogen *Puccinia striiformis* f. sp. *tritici* (*Pst*), is a serious disease of wheat in the United States and many other countries. Growing resistant cultivars has been approved to be the best approach for control of stripe rust. To determine stripe rust resistance genes in U.S. winter wheat cultivars and breeding lines, we analyzed a winter wheat panel of 857 cultivars and breeding lines in a genome-wide association study (GWAS) using genotyping by multiplexed sequencing (GMS) and by genotyping with molecular markers of 18 important stripe rust resistance genes or quantitative trait loci (QTL). The accessions were phenotyped for stripe rust response at adult-plant stage under natural infection in Pullman and Mount Vernon, Washington in 2018 and 2019, and in the seedling stage with six predominant or most virulent races of *Pst*. A total of 51 loci were identified to be related to stripe rust resistance, and at least 10 of them (*QYrww.wgp.1D-3*, *QYrww.wgp.2B-2*, *QYrww.wgp.2B-3*, *QYrww.wgp.2B-4*, *QYrww.wgp.3A*, *QYrww.wgp.5A*, *QYrww.wgp.5B*, *QYrww.wgp.5D*, *QYrww.wgp.6A-2* and *QYrww.wgp.7B-3*) were previously reported. These genes or QTL were found to be present at different frequencies in breeding lines and cultivars developed by breeding programs in various winter wheat growing regions. Both *Yr5* and *Yr15*, which are highly resistant to all races identified thus far in the U.S., as well as *Yr46* providing resistance to many races, were found absent in the breeding lines and commercially grown cultivars. The identified genes or QTL and their markers are useful in breeding programs to improve the level and durability of resistance to stripe rust.

## Introduction 

Stripe rust (yellow rust), caused by *Puccinia striiformis* Westend. f. sp. *tritici* Erikss. (*Pst*), is one of the most serious diseases threating wheat production in the world (Wellings, 2011; Ellis et al., 2014; Chen, 2020). In the United States, severe stripe rust epidemics have been recorded since 1958, but mostly in the western states. However, since the year 2000, the disease has caused nation-wide epidemics (Chen et al., 2002; Chen, 2005; Chen et al., 2010; Chen, 2020). From 1958 to 2018, stripe rust caused yield losses of more than 31,757,032 tons in the U.S. (Chen and Kang, 2017). Although stripe rust can be reduced by applying fungicides, the cost and potential environmental effects of using fungicides become a big concern (Chen, 2014; Chen, 2020). As an effective and sustainable approach, stripe rust should be controlled by growing resistant cultivars (McIntosh et al., 1995; Chen, 2005; Chen, 2013). Two types of resistance to stripe rust have been characterized and used in breeding programs. All-stage resistance (ASR), which can be detected in the seedling stage, is highly effective in all growth stages and easy to be phenotypically selected in breeding programs, but usually not durable as it can be circumvented by new virulent race of *Pst*. In contrast, adult-plant resistance (APR) expresses in late plant growth stages and the resistance level is often affected by temperature, and thus often called high-temperature adult-plant (HTAP) resistance. As APR or HTAP resistance usually provides partial resistance, this type of resistance may be inadequate (Chen, 2005; Chen, 2013; Chen, 2014; Ellis et al., 2014). However, APR or HTAP resistance is durable as it is usually not influenced much by *Pst* race changes (Chen, 2005; Chen, 2013). The best strategy is to combine APR or HTAP resistance with effective ASR to improve both level and durability of resistance.

To date, 85 formally named and more than 300 temporarily designated genes or quantitative trait loci (QTL) have been reported for stripe rust resistance (Maccaferri et al., 2015; Bulli et al., 2016; McIntosh et al., 2017; Wang and Chen, 2017). Unfortunately, many ASR resistance genes that have been widely deployed in wheat cultivars have been overcome by recently predominant races of *Pst*, and many minor effective QTL for APR are difficult to be used in breeding programs. Thus, it is still essential to identify new germplasms and genes for useful resistance to stripe rust. As stripe rust resistance genes in most breeding materials and commercially grown cultivars are not clear, it is also urgent to know which genes are in the currently grown cultivars and recently developed breeding lines. Such information should be useful for wisely deploying resistant cultivars based on their genes, as well as select effective and use diverse genes to develop new resistant cultivars.

In previous studies, most genes or QTL were identified using the classical bi-parental linkage mapping approach. This approach limits the number of the genetic stocks that can be tested in a study, takes years or intensive labor plus sophisticated doubled-haploid technique to develop a mapping population, and often has a low resolution in QTL detection (Flint-Garcia et al., 2003; Parisseaux and Bernardo, 2004). Recently, the genome-wide association study (GWAS) technique has emerged as an efficient approach for gene discovery (Maccaferri et al., 2015; Bulli et al., 2016; Godoy et al., 2018; Cheng et al., 2019). Using this approach, genes in hundreds to thousands of accessions can be determined simultaneously. For wheat, the high level of linkage disequilibrium can significantly reduce the number of markers needed for identifying marker-trait associations (MTAs) (Chao et al., 2010). However, the GWAS approach has several drawbacks. Population stratification often leads to spurious associations. Low frequent genes may not be identified because of either the limited statistic power (Myles et al., 2009) or being masked by a more frequent gene in the similar region (Liu et al., 2020). In order to discover more and robust MTAs, it is better to use a big large population and a set of markers tagging nonrepetitive genome regions, plus mixed linear models (MLMs) to eliminate spurious associations caused by population structure.

A large size of germplasm panel is ideal for GWAS, but genotyping may cost a lot. Because the genotyping cost, so far only few studies of wheat for stripe rust resistance have been conducted using large sizes of germplasm panels (Maccaferri et al., 2015; Bulli et al., 2016). Recently, a genotyping by multiplexed sequencing (GMS) platform has been established (Ruff et al., 2020). Currently consisting of 2,242 SNP markers that were carefully selected to cover the nonrepetitive genome regions of wheat, this platform is based on sequencing multiplex polymerase chain reaction (PCR) amplicons for genotyping. These SNP markers cover quite uniformly throughout wheat genome, especially tagging the nonrepetitive genome regions, with an average distance of 2.33 cM between markers on each chromosome. Using the GMS technique, we have successfully identified 37 genes or QTL for stripe rust resistance from 616 spring wheat cultivars and breeding lines developed in the U.S. (Liu et al., 2020).

In this study, we used the GMS technique and the GWAS approach to study stripe rust resistance in a winter wheat panel consisting of 857 accessions of genetic stocks, commercially grown cultivars, and advanced breeding lines developed and used in various wheat growing regions of the U.S. The objectives of the study were to determine genes for resistance to stripe rust in U.S. winter wheat and especially to identify genes not previously reported.

## Materials and Methods

### Plant Materials

The winter wheat panel of 857 cultivars and breeding lines was assembled from different U.S. regional nurseries of 2017 and genetic stock and stripe rust monitoring nurseries in our program. The accessions were from the following nurseries: (1) 163 accessions from the winter wheat cereal disease nurseries (1711_WCDN, consisting of international genetic stocks for monitoring *Pst* virulence changes and using in breeding); (2) 203 accessions from the winter wheat cultivar monitoring nursery (1709_WWCMN, consisting of cultivars recently grown in various states of the U.S.); (3) 185 accessions from the western regional disease nursery (1701_WRDN, consisting of winter wheat cultivars historically and recently developed and grown in the western U.S.); (4) 92 accessions from the winter extension disease nursery (1702_WEDN, consisting of commercially grown cultivars and advanced breeding lines in the western U.S.), (5) 20 accessions from the winter western uniform region nursery (1726_WURN consisting of breeding lines developed in the western U.S.); (6) 85 accessions from the winter hard wheat nursery (1712_WHWN, consisting of hard wheat breeding lines developed in the U.S. Great Plains); (7) 36 accessions from the winter eastern wheat nursery (1715_WEWN consisting of breeding lines in the eastern regional uniform nursery), (8) 30 accessions from the winter southern wheat nursery (1716_WSWN, consisting breeding lines in the southern regional uniform nursery, and (9) 43 accessions from the winter east stripe rust nursery (1718_WESR, consisting wheat lines developed in the eastern states). In addition, an experimental line, PS279 that is highly susceptible to stripe rust, was used as a susceptible check in the greenhouse and field tests and a spreader for increasing stripe rust pressure in the fields.

### Stripe Rust Phenotyping at the Seedling Stage in the Greenhouse

The 857 accessions were evaluated at the seedling stage for their infection type (IT) produced by six *Pst* races, PSTv-4, PSTv-14, PSTv-37, PSTv-40, PSTv-51, and PSTv-198, under controlled greenhouse conditions. These races are either the most virulent (PSTv-51) or recently predominant (the other five) in the U.S. (Wan and Chen, 2014; Wan et al., 2016; Liu et al., 2020). The virulence/avirulence formulae of these races are presented in **Table S1**. Our standard procedures were used for growing plants, inoculating and recording IT data (Chen and Line, 1992; Chen et al., 2002). Five seeds of each accession were planted. Seedlings were inoculated with a mixture of urediniospores and talc at a 1:20 ratio and incubated in a dew chamber at 10°C for 24 h in the darkness and grown in a growth chamber set at a diurnal cycle changing from 4°C at 2:00 am to 20°C at 2:00 pm and 8-h dark/16-h light (Chen and Line, 1992). IT data based on the 0–9 scale were recorded 18–20 days after inoculation when stripe rust was fully developed on the susceptible check (Line and Qayoum, 1992). The IT data were used for GWAS analysis.

### Stripe Rust Phenotyping at the Adult-Plant Stage in Fields

The winter wheat panel was evaluated under natural infection of *Pst* in four environments: Mount Vernon (48°259’1299N, 122°199 3499W) in the northwest and Pullman (46°43’ 5999N, 117°109’0099W) in the southeast of Washington state in 2018 and 2019. The two sites are about 500 km apart and have different weather conditions and different *Pst* race compositions (Liu et al., 2020). In all field trials, the accessions were planted in October 2017 and October 2018 as nonreplicated single rows with about 5-g seed planted in each row for stripe rust phenotyping in 2018 and 2019. Rows were 0.5-m long with a space of 0.25 m between rows. The susceptible check PS279 was planted every 20 rows and also surrounding the field to allow stripe rust developing to the uniform and highest level. Infection type (IT) and disease severity (DS) were recorded between heading (Zadoks Growth Stage (GS) 50 (Zadoks et al., 1974) when flag leaves of the susceptible check plants had at least 50% DS and soft dough (GS 70) stages when most flag leaves of the susceptible check plants had at more than 95% DS. The IT data were scored using a 0–9 scale as described previously (Line and Qayoum, 1992), and the DS data were scored as percentage of infected leaf area. Both IT and DS data of the second record were used for GWAS analyses.

### Phenotypic Data Analysis

Analysis of variance (ANOVA) was conducted using SAS V8.0 (SAS Institute, Cary, NC, USA) to determine effects of genotypes, environments (location and year), and the interactions between genotypes and environments. Broad-sense heritability (H^2^) was estimated using the variance components from the ANOVA model. The best linear unbiased estimator (BLUE) values were calculated across the different environments considering genotypes as a fixed effect in the model using software QTL IciMapping (http://www.isbreeding.net). The BLUE values were also used for the GWAS analyses.

### Genotyping

For each accession, the third leaves at the seedling stage were collected for DNA extraction. Genomic DNA was extracted in the same way as described in Liu et al. (2020). The 857 accessions were genotyped using GMS as previously described (Liu et al., 2020; Ruff et al., 2020). To overcome the drawback of GWAS unable to detect rare alleles, we also used molecular markers for 18 important genes or QTL for stripe rust resistance to test the accessions. A total of 31 simple-sequence repeat (SSR), sequence-tagged site (STS), or kompetitive allele specific PCR (KASP) markers were used for determining the presence or absence of the following previously reported 18 *Yr* (yellow rust) genes or QTL in the accessions: *Yr5* (Marchal et al., 2018), *Yr9* (Mago et al., 2002), *Yr10* (Bariana et al., 2002), *Yr15* (Ramirez-Gonzalez et al., 2015), *Yr17* (Helguera et al., 2003), *Yr18* (Lagudah et al., 2009), *Yr27* (Chhetri et al., 2017), *Yr30* (Spielmeyer et al., 2003), *Yr46* (Forrest et al., 2014), *Yr76* (Xiang et al., 2016), *Yr78* (Dong et al., 2017), *YrSP* (Feng et al., 2015), *YrTr1* (X. M. Chen and associates, unpublished), *QYrMa.wgp-1AS* (Liu et al., 2018), *QYrel.wgp-2BS* (Liu et al., 2019a), *QYrsk.wgp-3BS* (Liu et al., 2019b), *QYrsk.wgp-4BL* (Liu et al., 2019b), and *QYr.wpg-1B.1* (Naruoka et al., 2015). DNA samples from spring wheat lines carrying specific *Yr* genes, which were used in the previous GWAS study with spring wheat (Liu et al., 2020), were included as positive controls for the genotyping with molecular markers, but their data were excluded from the data analysis of the winter wheat panel. PCR amplifications of SSR markers representing *Yr9*, *Yr10*, *Yr17*, *Yr27*, *Yr30*, *Yr76*, *Yr78*, *YrSP*, and *YrTr1* were based on the information of GrainGenes and MAS Wheat (http://wheat.pw.usda.gov, http://maswheat.ucdavis.edu). PCR products were detected using an ABI3730 DNA fragment analyzer (Applied Biosystems, Grand Island, NY, USA), and the alleles were scored using software GeneMarker v4.0 (SoftGenetics, LLC, State College, PA, USA), as described in the previous study (Liu et al., 2020). KASP markers representing *Yr5*, *Yr15*, *Yr18*, *Yr46*, *QYrMa.wgp-1AS*, *QYrel.wgp-2BS*, *QYrsk.wgp-3BS*, *QYrsk.wgp-4BL*, and *QYr.wpg-1B.1* were obtained from the studies by Liu et al. (2018; 2019a; 2019b; 2020). KASP markers were tested as described by Liu et al. (2019b; 2020), and the end-point fluorescence data were visualized and recorded using a Roche Light-Cycler 480 real-time PCR system (Roche Applied Science, Indianapolis, IN, USA). The primer sequences of these markers are given in **Table S2**.

### Analyses of Population Structure

The population structure of the winter wheat panel was analyzed using Bayesian model-based clustering and distance-based hierarchical clustering in software STRUCTURE v.2.3.4 (Pritchard et al., 2000). An admixture model of population structure based on correlated allele frequencies was used to run five independent iterations for each subpopulation (settings from 1 to 10). In each time of iteration, a 10,000 length burn-in period was used and after burn-in, 10,000 Markov Chain Monte Carlo (MCMC) replications were conducted. STRUCTURE outputs were collated using the web-based software STRUCTURE HARVEST (Earl and Vonholdt, 2012; http://taylor0.biology.ucla.edu/structureHarvester/). The optimum subpopulation numbers were selected using the *ad hoc* △k statistic method (Evanno et al., 2005). To generate the population structure matrix (Q), the output of STRUCTURE HARVEST was imported into the program Clumpp v1.1.2 (Jakobsson and Rosenberg, 2007). Software Distruct v1.1 Q was used to develop bar graphs, and Tassel 2.3 (Rosenberg, 2004) was used to determine the panel kinship.

### Linkage Disequilibrium

Software HAPLOVIEW v4.2 was used to estimate the LD squared allele frequency correlation (*r* ^2^) for all pairwise comparisons between SNPs on the same chromosome and to visualize the local LD patterns (Barrett et al., 2005). To visualize the overall pattern of LD decay over genetic distances, syntenic pairwise LD *r* ^2^ estimates from all chromosomes were plotted against the corresponding pairwise genetic distances. The R program based on the equation relating LD, recombination rate, and population size was used to fit a nonlinear regression model (Sved, 1971; Rexroad and Vallejo, 2009). To define the confidence intervals of QTL in the present study, the map distance at which LD fell below the *r* ^2^ threshold of 0.2 was used, as the LD threshold have been frequently used for detecting QTL (Ardlie et al., 2002; Shifman et al., 2003; Khatkar et al., 2008; Lawrence et al., 2009).

### Genome-Wide Association Analyses

Markers with greater than 50% missing data were filtered out, and the remaining marker data were imputed using BEAGLE 3.3.2 (Browning and Browning, 2016). Software GAPIT in the R package (Lipka et al., 2012) was used to analyze associations between SNP markers and stripe rust reaction data. To reduce possible spurious associations caused by population structure, a MLM with Q and K as covariates (Yu et al., 2005) was used in the GWAS analyses of the present study. Markers with *P* < 0.001 were considered significantly associated to the stripe rust phenotype. Significant markers were assigned to QTL based on their chromosomal positions. Software CMplot in the R package (https://github.com/YinLiLin/R-CMplot) was used to drawn Manhattan plots.

### Determining Resistance Gene/QTL Frequencies

The tagging marker or markers were used to determine the presence or absence of each stripe rust gene or QTL in the 857 winter wheat accessions. To reduce false positives, accessions that had IT or DS values similar to that of the susceptible check were considered not to have the resistance gene or QTL, even though they have the positive resistance marker allele (Liu et al., 2020). The frequencies of each stripe rust resistance gene/QTL were calculated for the different nurseries and the whole panel.

### Determining the Effects of Different Numbers of Resistance Alleles

Individual markers or haplotypes of two or more markers of individual resistance locus were used to determine the effects of different numbers of resistance alleles on stripe rust phenotypes. The alleles that reduced the disease scores were considered resistance alleles. A linear model regression was applied to the phenotype and number of resistance alleles in each accession. For ASR detected in the greenhouse seedling tests, the mean IT value of all race tests was regressed against the numbers of resistance alleles. For resistance detected in the fields, both mean IT and DS values of all environments were regressed against the numbers of resistance alleles identified from the field data.

### Determination of Relationships of the Identified QTL With Previously Reported Genes or QTL

To determine the relationships with previously reported genes or QTL for stripe rust resistance (Maccaferri et al., 2015; Bulli et al., 2016; Wang and Chen, 2017; Liu et al., 2020), all resistance genes or QTL identified in the present study were placed in the integrated genetic map based on their marker locations. The physical locations of the markers were determined through Basic Local Alignment Search Tool (BLAST) search of the hexaploid wheat reference genome (IWGSC RefSeq v.1.1). BLAST hits were selected using the 10^−5^ e-value threshold and a minimum similarity cutoff >95% between the query and database sequences.

## Results

### Phenotypic Distribution

**Figure 1:**
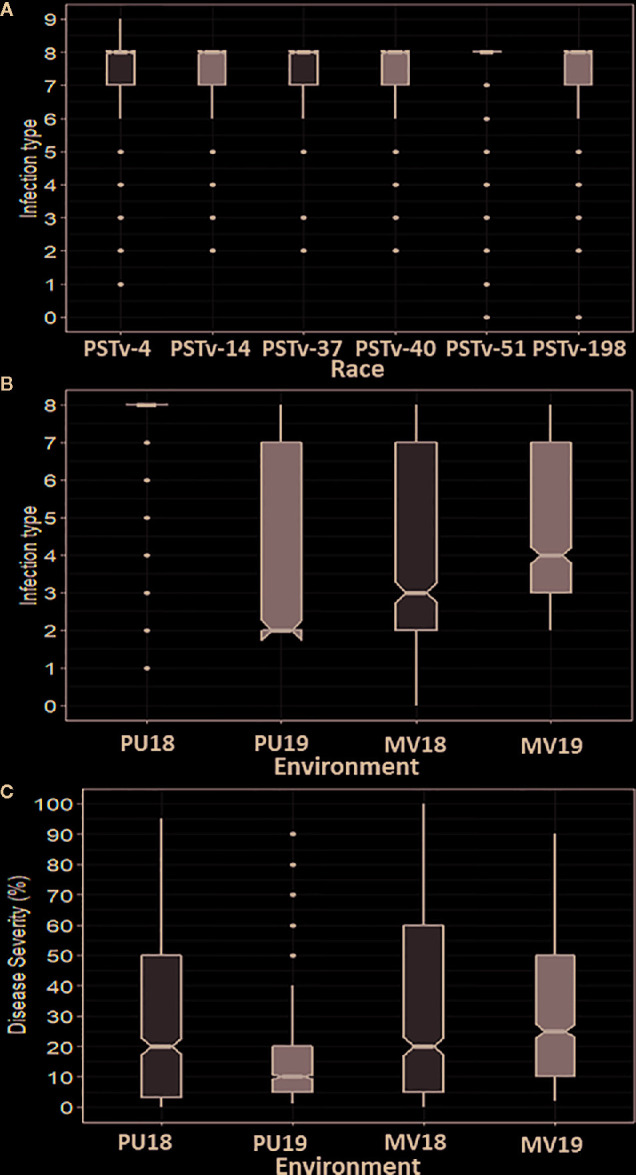
Distributions of stripe rust infection type (IT) and disease severity (DS). **(A)** IT distributions in the seedling tests with different races of *Puccinia striiformis* f. sp. *tritici*. **(B)** IT distribution across field environments including Pullman 2018 (Pu18), Mount Vernon 2018 (MV18), Pullman 2019 (Pu19), and Mount Vernon 2019 (MV19). **(C)** DS distribution across field environments. Solid horizontal lines display the median values. Notches display a 95% confidence interval around the median. Top and bottom box edges display the first and third quartile values, respectively. Whiskers display the largest and smallest values within 1.5 times the interquartile range. Dots represent the values out of 1.5 times the interquartile range.

The IT data of the seedlings tested with the six *Pst* races and IT and DS data of the adult-plant stage evaluated in the four field environments for the 857 winter wheat accessions are given in **Table S3**. The distributions of IT and SEV scores recorded in the greenhouse seedling tests and the field environments are shown in **Figure 1**. The seedling responses to stripe rust skewed toward high IT (susceptible reaction), with 12%, 10%, 8%, 11%, 10%, and 9% of the accessions showing resistant reactions (0–3); 2%, 7%, 2%, 3%, 1%, and 1% displayed intermediate resistant reactions (4–6); and 86%, 83%, 90%, 87%, 89%, and 90% were susceptible (7–9) in the tests with PSTv-4, PSTv-14, PSTv-37, PSTv-40, PSTv-51, and PSTv-198, respectively (**Figure 1A**). In contrast, the stripe rust responses in the field tests were close to a normal distribution. A wide range of variation was observed in the panel across all environments, ranging from very resistant to very susceptible. The susceptible check PS279 displayed consistently high susceptibility with IT 8–9 and DS >95% in all environments. The mean IT within environments ranged from 3.8 to 4.5, and the mean DS ranged from 21.0 to 31.8% (**Figures 1B, C**). Significant correlations (*P* < 0.001) were observed between IT and DS (*r*: 0.83 to 0.94) in the same environments and for IT (*r*: 0.73 to 0.88) and DS (*r*: 0.78 to 0.91) between different environments (**Table 1**), indicating the relative consistency of *Pst* responses. For both IT and DS, the correlation coefficients were slightly higher between different years in the same location than between different locations in the same years. The correlations between IT and DS at the same years in Mount Vernon were slightly higher than those in Pullman. Broad-sense heritability H^2^ was 0.85 for IT and 0.74 for DS. The more susceptible reactions in the seedling tests at low temperature and more resistant reactions at the adult-plant stage in the fields indicated that many accessions in the panel had APR or HTAP resistance.

**Table 1:** Correlation coefficients (*r*) of stripe rust infection type (IT) and disease severity (DS) across four environments.

Environment^a^	Correlation coefficient (*r*)^b^			
	PU18	MV18	PU19	MV19
PU18	**0.83**	0.78	0.88	0.78
MV18	0.83	**0.94**	0.74	0.88
PU19	0.91	0.78	**0.81**	0.73
MV19	0.82	0.85	0.80	**0.92**

^a^The four environments were field tests in Pullman 2018 (PU18), Mount Vernon 2018 (MV18), Pullman 2019 (PU19), and Mount Vernon 2019 (MV19) under natural infection.

^b^r values calculated between IT and DS in the same environment are presented in the diagonal line (in bold), and those with IT in the right top half and those with DS in the left bottom half are between different environments. All r values are significant at P < 0.001.

### GMS Markers and Population Structure

After eliminating monomorphic markers and filtering out markers with 50% or more missing data, 1,588 polymorphic markers were obtained from the GMS genotyping. These markers were used in population structure, LD, and GWAS analyses.

**Figure 2:**
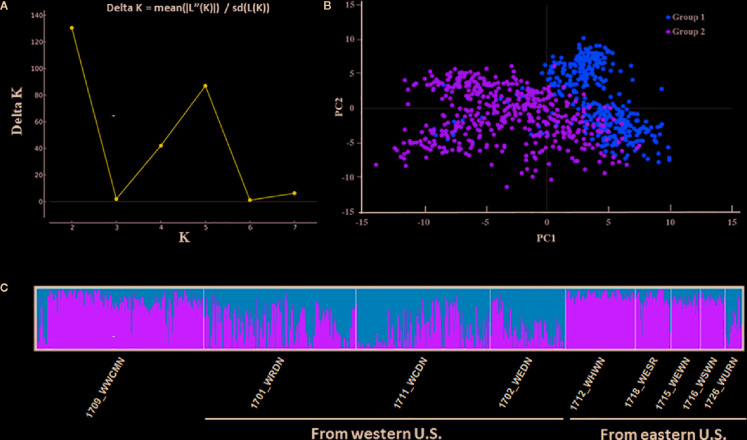
Model-based population structure of the 857 winter wheat accessions combined with markers. **(A)** The result obtained from Structure Harvester analysis (k = 2). **(B)** The STRUCTURE analysis showed two hypothetical subpopulations represented by different colors. **(C)** First two components (PC1 and PC2) of a principal component analysis.

Two subpopulations were found to be in the winter wheat panel of 857 accessions (**Figure 2A**). An evident differentiation between both groups was shown in the STRUCTURE analysis. Group 1 consisting of 460 accessions marked in yellow had a higher proportion of the population, and Group 2 had a smaller number (397) accessions marked in green (**Figure 2B**). Accessions in Group 1 were from nurseries 1701_WRDN (40%), 1702_WEDN (20%), 1711_WCDN (35%), and 1726_WURN (4%), which were mainly from the western U.S., while accessions in Group 2 were from nurseries 1709_WWCMN (51%), 1712_WHWN (21%), 1715_WEWN (9%), 1716_WSWN (8%), and 1718_WESR (11%), mainly from the eastern U.S. except some cultivars from the western U.S. in 1709_WWCMN. Group 1 had lower disease scores than Group 2 in both greenhouse seedling and field tests (*P* < 0.001), indicating relatively more resistance of Group 1. The separation of two subgroups was supported by the principal component analyses (PCA, **Figure 2C**). Accessions from Group 1 and Group 2 in the same region of PC2 were differentiated only by PC1.

### Linkage Disequilibrium

**Figure 3:**
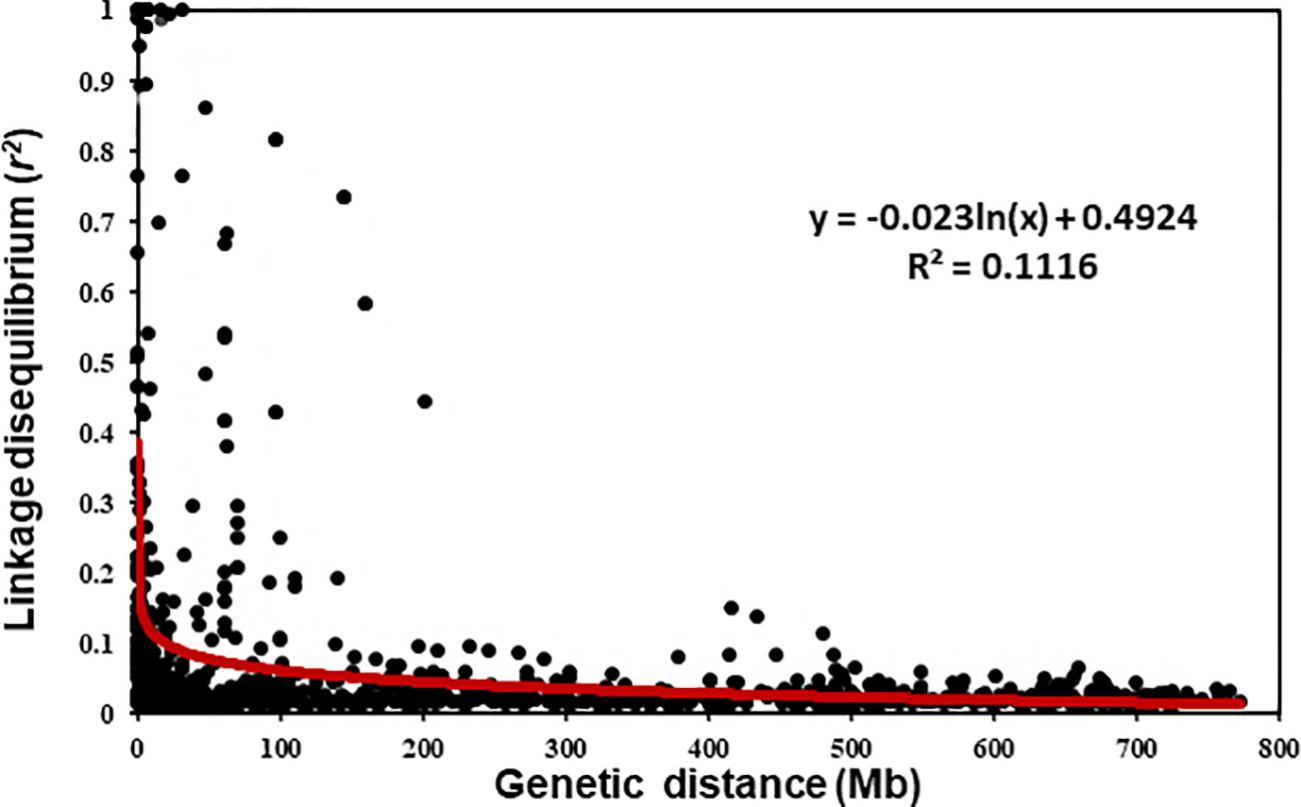
Overview of the linkage disequilibrium (LD) parameter *R* ^2^ of the intra-chromosomal pairs in the winter wheat panel.

The LD *r* ^2^ values between the polymorphic SNP markers on each chromosome were estimated. The scatter plot of LD values against the physical distance is shown in **Figure 3**. The LD decayed to the critical *r* ^2^ value (0.2) was estimated at about 17 Mb for the entire genome. Thus, markers within 17 Mb or the *r* ^2^ value between the markers greater than 0.2 were considered to represent the same QTL with few exceptions on some D chromosomes that had relatively low numbers of markers. For the exceptions, the differences in resistance-allele frequencies of the winter wheat panel were used to determine whether the markers represent different loci.

### QTL for ASR to Stripe Rust Determined by GWAS Using the Greenhouse Seedling Data

**Figure 4:**
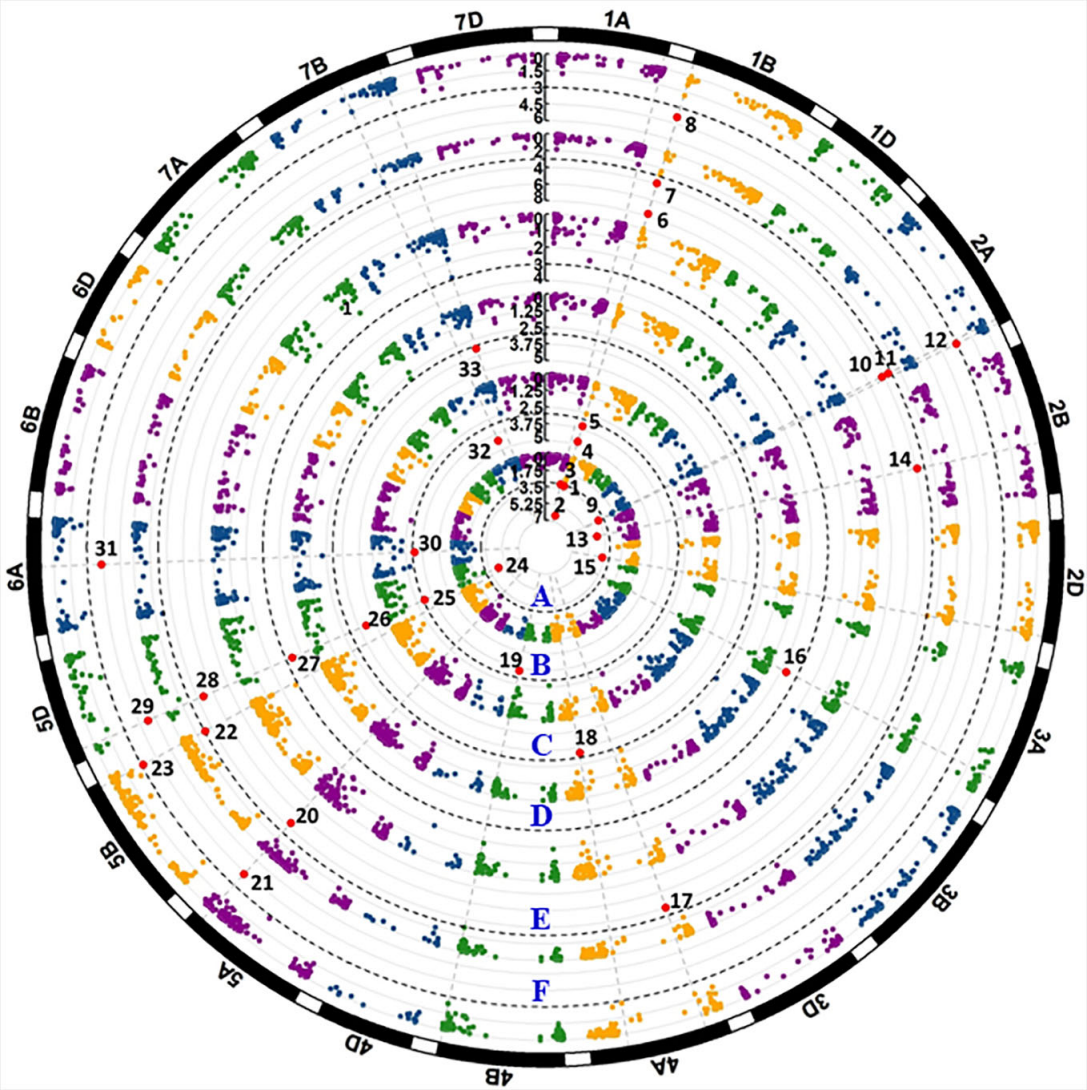
Manhattan plots of significant *p* values for markers associated with all-stage resistance to stripe rust. The black dash line shows the threshold -log10(p) value of 3. Each circular represents one seedling test with race PSTv-4 **(A)**, PSTv-14 **(B)**, PSTv-37 **(C)**, PSTv-40 **(D)**, PSTv-51**(E)**, and PSTv-198 **(F)**. Significant markers are enlarged in red dots and numbered.

The IT data of the 857 accessions tested with races PSTv-4, PSTv-14, PSTv-37, PSTv-40, PSTv-51, and PSTv-198 (**Table S3**) were used to identify QTL for ASR. A total of 16 markers, representing 15 QTL, were significantly associated with ASR, including 1 QTL each on 1A, 2A, 1B, 2B, 2D, 3A, 4B, 5A, 5B, 5D, and 6A; and 2 QTL each on 4A and 7B. QTL *QYrww.wgp.2A-2* was represented by two markers (*IWB25290* and *IWB6997*) at the positions of 737 and 739 Mb on 2AL, respectively (**Table 2** and **Figure 4**). The presence and absence of the resistance alleles of the markers in the 857 accessions are given in **Table S4**. Eight of the 15 QTL, *QYrww.wgp.1A-3* (No. 1 to 2), *QYrww.wgp.2D-4* (No. 15), *QYrww.wgp.3A* (No. 16), *QYrww.wgp.4A-1* (No. 17), *QYrww.wgp.4A-2* (No. 18), *QYrww.wgp.4B* (No. 19), *QYrww.wgp.7B-2* (No. 32), and *QYrww.wgp.7B-3* (No. 33), were resistant to only one race. The remaining seven of the 15 ASR QTL, *QYrww.wgp.1B-1* (indicated by Nos. 3 to 8 in **Figure 4**), *QYrww.wgp.2A-2* (No. 10-12), *QYrww.wgp.2B-4* (No. 14), *QYrww.wgp.5A* (Nos. 20 to 21), *QYrww.wgp.5B* (No. 22 to 23), *QYrww.wgp.5D* (No. 24 to 29), and *QYrww.wgp.6A-1* (No. 30 to 31), were resistant to two or more races. Two QTL, *QYrww-wgp.1B* and *QYrww-wgp.5D*, were effective against all six tested races. The phenotypic variation (*R* ^2^) values of the ASR QTL explained ranged between 0.08 and 0.26 (**Table 2**).

**Table 2:** Resistance quantitative trait loci (QTL) identified in the winter wheat panel associated with stripe rust reactions evaluated at the seedling stage in the greenhouse with different races of *Puccinia striiformis* f. sp. *tritici*.

QTL	Race	SNP	Chr^a^	Position (Mb)^a^	-log10(p)	MAF^b^	R^2^ ^c^	Alleles^d^
*QYrww.wgp.1A-3*	PSTv-4	IWA3409	1AL	571	3.01	0.08	0.11	G/**A**
*QYrww.wgp.1B*	PSTv-4	IWB44699	1BS	2	3.91	0.15	0.12	**G**/A
	PSTv-14	IWB44699			4.32	0.15	0.11	**G**/A
	PSTv-37	IWB44699			3.05	0.15	0.11	**G**/A
	PSTv-40	IWB44699			4.09	0.15	0.12	**G**/A
	PSTv-51	IWB44699			3.09	0.15	0.11	**G**/A
	PSTv-198	IWB44699			3.82	0.15	0.12	**G**/A
*QYrww.wgp.2A-2*	PSTv-51	IWB25290	2AL	737	3.34	0.06	0.11	G/**A**
	PSTv-51	IWB6997	2AL	739	4.19	0.10	0.09	G/**A**
	PSTv-4	IWB6997			3.57	0.10	0.12	G/**A**
								
*QYrww.wgp.2B-4*	PSTv-4	IWB34793	2BL	524	4.25	0.05	0.11	G/**A**
	PSTv-51	IWB34793			3.88	0.05	0.12	G/**A**
*QYrww.wgp.2D-4*	PSTv-4	IWA229	2DL	619	3.72	0.06	0.23	A/**G**
*QYrww.wgp.3A*	PSTv-40	IWB44443	3AL	639	3.68	0.07	0.18	G/**A**
*QYrww.wgp.4A-1*	PSTv-37	IWB44966	4AL	538	3.36	0.24	0.25	**A**/G
*QYrww.wgp.4A-2*	PSTv-51	IWA5116	4AL	605	3.22	0.20	0.14	**C**/A
*QYrww.wgp.4B*	PSTv-14	IWA2869	4BL	666	3.52	0.09	0.13	G/**A**
*QYrww.wgp.5A*	PSTv-51	IWB43581	5AL	644	4.50	0.11	0.22	G/**T**
	PSTv-198	IWB43581			4.32	0.11	0.24	G/**T**
*QYrww.wgp.5B*	PSTv-51	IWB29509	5BL	685	3.09	0.14	0.19	G/**A**
	PSTv-198	IWB29509			3.16	0.14	0.25	G/**A**
*QYrww.wgp.5D*	PSTv-4	IWB16856	5DS	59	4.44	0.11	0.15	A/**G**
	PSTv-14	IWB16856			3.09	0.11	0.21	A/**G**
	PSTv-37	IWB16856			4.30	0.11	0.13	A/**G**
	PSTv-40	IWB16856			3.38	0.11	0.11	A/**G**
	PSTv-51	IWB16856			4.73	0.11	0.12	A/**G**
	PSTv-198	IWB16856			5.28	0.11	0.12	A/**G**
*QYrww.wgp.6A-1*	PSTv-14	IWB52712	6AL	356	3.16	0.43	0.11	T/**C**
	PSTv-198	IWB52712			4.31	0.43	0.09	T/**C**
*QYrww.wgp.7B-2*	PSTv-14	IWA6322	7BL	457	4.25	0.20	0.24	G/**A**
*QYrww.wgp.7B-3*	PSTv-37	IWB72939	7BL	683	3.21	0.14	0.26	G/**A**

a Chromosomes and positions of markers were determined according to IWGSC RefSeq v.1.1.

b MAF, minor allele frequency.

c R^2^ indicated phenotypic variance explained by the associated marker.

d Resistance-associated allele was labeled in bold and underlined character.

### QTL for Stripe Rust Resistance Determined by GWAS Using the Field Adult-Plant Data

**Figure 5:**
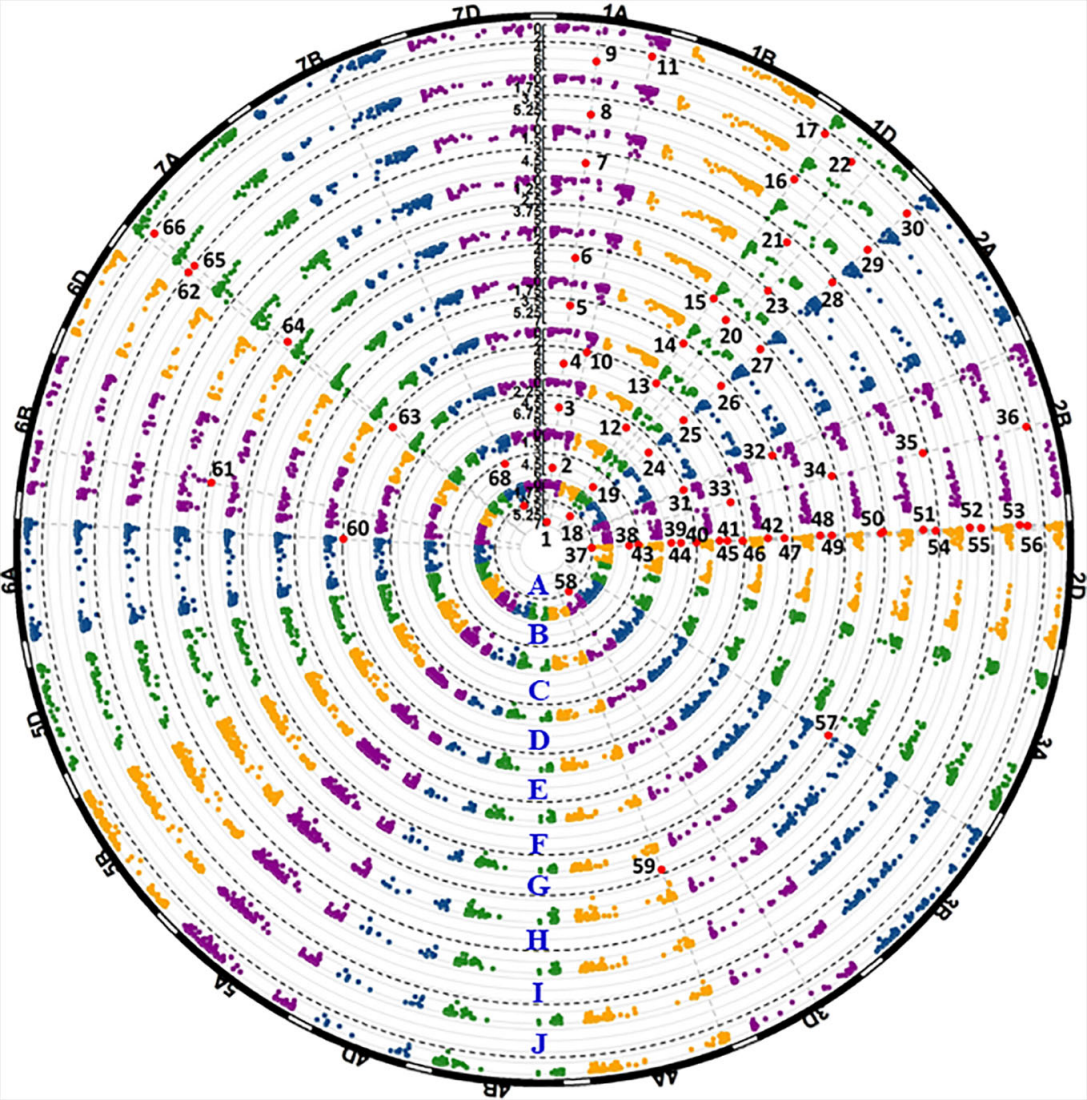
Manhattan plots of significant *p* values for markers associated with stripe rust resistance detected in the field experiments. The black dash lines show the threshold -log10(p) value of 3. Each circular represents one field data set of Pullman 2018 IT data **(A)**, Pullman 2018 DS data **(B)**, Mount Vernon 2018 IT data **(C)**, Mount Vernon 2018 DS data **(D)**, Pullman 2019 IT data **(E)**, Pullman 2019 DS data **(F)**, Mount Vernon 2019 IT data **(G)**, Mount Vernon 2019 DS data **(H)**, BLUE-ALL-IT data **(I)**, and BLUE-ALL-DS data **(J)**. Significant markers are enlarged in red dots and numbered.

A total of 20 significantly associated markers (*P* < 0.001) representing 20 QTL were detected (**Table 3**, **Figure 5**, and **Table S4**). Three QTL were detected by both IT and DS data. *QYrww.wgp.1A-1* (indicated by No. 1 to 9 in **Figure 5**) was identified on the long arm of chromosome 1A (1AL) by *IWB8633* at the 224 Mb position. This QTL was significant for both IT and DS from the tests in Pullman in 2018 and 2019 (PU18, PU19) and Mount Vernon in 2018 and 2019 (MV18, MV19). The *R* ^2^ value of *QYrww.wgp.1A-1* for IT and DS ranged from 0.08 to 0.12 and 0.09 to 0.12, respectively. *QYrww.wgp.1D-1* (No. 12 to 17) was identified by *IWB2803* at the 4 Mb position on the short arm of 1D (1DS) with a *R* ^2^ value ranging from 0.10 to 0.15 and 0.08 to 0.10 for IT and DS, respectively. This QTL was significant for IT in PU18, MV18, PU19, and MV19, and DS evaluated from PU18 and MV19. *QYrww.wgp.1D-3* (No. 23) was linked to *IWA3446* located at 203 Mb on the long arm of 1D (1DL). This QTL was significant for DS in PU18, MV19, and PU19 and IT in MV18. The *R* ^2^ values of this QTL for both IT and DS ranged between 0.08 and 0.12.

**Table 3:** Resistance genes or QTL identified in the winter wheat panel associated with stripe rust reactions evaluated at the adult-plant stage in field environments under natural infection of *Puccinia striiformis* f. sp. *tritici*.

Gene/QTL	Traits^a^	SNP	Chr^b^	Position (Mb)^b^	-log10(p)	MAF^c^	R^2^ ^d^	Alleles^e^
*QYrww.wgp.1A-1*	PU18-IT	IWB8633	1AL	223	3.48	0.07	0.08	A/**C**
	PU18-DS	IWB8633			4.13	0.11	0.12	A/**C**
	MV18-IT	IWB8633			4.02	0.13	0.10	A/**C**
	MV18-DS	IWB8633			4.48	0.11	0.12	A/**C**
	PU19-IT	IWB8633			4.62	0.10	0.11	A/**C**
	PU19-DS	IWB8633			5.04	0.11	0.09	A/**C**
	MV19-DS	IWB8633			4.96	0.08	0.09	A/**C**
	BLUE-IT-ALL	IWB8633			3.98	0.15	0.12	A/**C**
	BLUE-DS-ALL	IWB8633			5.33	0.09	0.13	A/**C**
*QYrww.wgp.1A-2*	MV18-DS	IWA6710	1AL	518	3.09	0.33	0.11	**T**/G
	MV19-DS	IWA6710			3.29	0.33	0.09	**T**/G
*QYrww.wgp.1D-1*	MV18-IT	IWB2803	1DS	4	3.23	0.10	0.15	**T**/G
	MV18-DS	IWB2803			3.28	0.08	0.11	**T**/G
	PU19-IT	IWB2803			4.18	0.10	0.11	**T**/G
	PU19-DS	IWB2803			3.02	0.11	0.14	**T**/G
	BLUE-IT-ALL	IWB2803			3.31	0.10	0.13	**T**/G
	BLUE-DS-ALL	IWB2803			3.65	0.12	0.09	**T**/G
*QYrww.wgp.1D-2*	MV19-IT	IWA1787	1DS	8	4.45	0.10	0.11	**C**/G
*QYrww.wgp.1D-3*	MV18-IT	IWA3446	1DL	203	4.79	0.44	0.10	**G**/T
	PU18-DS	IWA3446			3.91	0.44	0.11	**G**/T
	PU19-DS	IWA3446			4.82	0.44	0.08	**G**/T
	MV19-DS	IWA3446			4.02	0.44	0.12	**G**/T
	BLUE-DS-ALL	IWA3446			5.23	0.44	0.11	**G**/T
*QYrww.wgp.2A-1*	PU19-IT	IWB40903	2AS	4	4.39	0.25	0.12	G/**A**
	PU19-DS	IWB40903			4.39	0.25	0.11	G/**A**
	MV18-IT	IWB40903			4.72	0.25	0.12	G/**A**
	MV18-DS	IWB40903.2A			4.72	0.25	0.10	G/**A**
	MV19-DS	IWB40903			3.62	0.25	0.12	G/**A**
	BLUE-IT-ALL	IWB40903			4.46	0.25	0.11	G/**A**
	BLUE-DS-ALL	IWB40903			4.46	0.25	0.14	G/**A**
*QYrww.wgp.2B-1*	MV18-IT	IWB47291	2BS	9	3.10	0.46	0.15	**C**/T
*QYrww.wgp.2B-2*	PU19-IT	IWB54530	2BS	47	3.33	0.12	0.11	**A**/G
*QYrww.wgp.2B-3*	MV18-DS	IWA3452	2BS	420	3.73	0.14	0.13	**C**/A
	PU19-DS	IWA3452			4.00	0.14	0.10	**C**/A
	MV19-DS	IWA3452			3.65	0.14	0.11	**C**/A
	BLUE-DS-ALL	IWA3452			4.58	0.14	0.14	**C**/A
*QYrww.wgp.2D-1*	PU18-IT	IWB22615	2DS	5	8.90	0.29	0.12	**C**/T
	PU18-DS	IWB22615			3.82	0.29	0.14	**C**/T
	MV19-IT	IWB22615			6.84	0.29	0.13	**C**/T
	MV19-DS	IWB22615			9.23	0.29	0.13	**C**/T
	BLUE-IT-ALL	IWB22615			5.15	0.29	0.12	**C**/T
	BLUE-DS-ALL	IWB22615			7.41	0.29	0.14	**C**/T
*QYrww.wgp.2D-2*	PU18-IT	IWB57369.2D	2DS	12	5.97	0.28	0.17	C/**T**
	PU18-DS	IWB57369.2D			4.25	0.28	0.20	C/**T**
	MV18-DS	IWB57369			4.25	0.28	0.11	C/**T**
	MV18-IT	IWB57369			7.11	0.28	0.18	C/**T**
	MV19-DS	IWB57369			7.11	0.28	0.12	C/**T**
	BLUE-IT-ALL	IWB57369			3.47	0.28	0.16	C/**T**
	BLUE-DS-ALL	IWB57369			3.47	0.28	0.17	C/**T**
*QYrww.wgp.2D-3*	PU18-IT	IWB5467	2DS	20	3.99	0.08	0.09	C/**A**
	PU19-DS	IWB5467			3.05	0.08	0.11	C/**A**
	MV19-IT	IWB5467			3.51	0.08	0.13	C/**A**
	MV19-DS	IWB5467			3.03	0.08	0.12	C/**A**
*QYrww.wgp.3B*	MV19-IT	IWB8118	3BL	796	4.92	0.06	0.10	C/**A**
*QYrww.wgp.3D*	PU18-IT	IWA3531	3DS	26	3.45	0.09	0.11	**C**/A
*QYrww.wgp.4A-3*	MV19-IT	IWA559	4AL	699	4.02	0.23	0.08	A/**G**
*QYrww.wgp.6A-2*	MV18-DS	IWA214	6AL	600	3.66	0.50	0.09	**T**/C
*QYrww.wgp.6B*	MV19-IT	IWA1017	6BL	528	4.01	0.23	0.08	A/**G**
*QYrww.wgp.7A-1*	MV18-DS	IWB49467	7AS	57	3.36	0.06	0.15	**T**/C
	PU19-DS	IWB49467			4.49	0.06	0.23	**T**/C
	BLUE-IT-ALL	IWB49467			4.48	0.06	0.18	**T**/C
	BLUE-DS-ALL	IWB49467			3.44	0.06	0.14	**T**/C
*QYrww.wgp.7A-2*	BLUE-IT-ALL	IWB9609	7AS	19	3.10	0.09	0.13	G/**T**
*QYrww.wgp.7B-1*	MV18-IT	IWA4362	7BL	435	3.16	0.08	0.09	C/**T**
	MV19-DS	IWA4362			3.35	0.08	0.11	C/**T**

a The winter wheat panel was evaluated at the adult-plant stage in the fields at Pullman and Mount Vernon in 2017 and 2018, referred as PU17, PU18, MV17, and MV18, respectively.

b Chromosomes and positions of markers were determined according to IWGSC RefSeq v.1.1.

c MAF, minor allele frequency.

d R^2^ indicated phenotypic variance explained by the associated marker.

e Resistance-associated allele was labeled in bold and underlined character.

Eight QTL located on chromosomes 1A, 1D, 2A, 2B, 2D, 7A, and 7B were detected in at least two environments, although not in all environments. *QYrww.wgp.1A-2* (No. 10 to 11 in **Figure 5**) was also identified by *IWA6710* at 518 Mb also on 1AL. This QTL was only significant for the DS data recorded in the Mount Vernon tests. The *R* ^2^ of *QYrww.wgp.1A-2* for DS ranged from 0.09 to 0.11. *QYrww.wgp.2A-1* (No. 24 to 30) was identified by *IWB40903* at 4 Mb on the long arm of 2A (2AL) with an *R* ^2^ ranging from 0.12 to 0.16 and 0.10 to 0.13 for IT and DS, respectively. This QTL was significant in all environments for IT and DS except for PU18. *QYrww.wgp.2B-3* (No. 33 to 36) was identified by *IWA3452* at 420 Mb on the long arm of 2B (2BL). This QTL was significant only for the DS data evaluated from all tested environments with *R* ^2^ ranging from 0.10 to 0.16. Three QTL, *QYrww.wgp.2D-1* (No. 38 to 43), *QYrww.wgp.2D-2* (No. 44 to 50), and *QYrww.wgp.2D-3* (No. 51 to 56), were identified on the short arm of 2D (2DS). These QTL were significant for the IT and DS data of different environments with *R* ^2^ values ranging from 0.09 to 0.23. *QYrww.wgp.7A-1* (No. 63 to 66) was identified on the short arm of 7A (7AS). This QTL was significant for both IT and DS data from MV18 and PU19 with *R* ^2^ ranging from 0.15 to 0.23. *QYrww.wgp.7B-1* (No. 67 to 68) mapped to the long arm of 7B (7BL) was significant for IT in 2018 and DS in 2019 at only Mount Vernon with *R* ^2^ values of 0.09 to 0.11, respectively.

The remaining nine QTL were detected with only one set (either IT or DS) of the field data. These QTL included *QYrww.wg.1D-2* (No. 23) on the short arm of 1D (1DS), *QYrww.wgp.2B-1* (No. 31) on the short arm of 2B (2BS), *QYrww.wgp.2B-2* (No. 32) on 2BS, *QYrww.wgp.3B* (No. 57) on the long arm of 3B (3BL), *QYrww.wgp.3D* (No. 58) on the short arm of 3D (3DS), *QYrww.wgp.4A-3* (No. 59) on the long arm of 4A (4AL), *QYrww.wgp.6A-2* (No. 60) on the long arm of 6A (6AL), *QYrww.wgp.6B* (No. 61) on the long arm of 6B (6BL), and *QYrww.wgp.7A-2* (No. 62) on 7AS. The *R* ^2^ values ranged from 0.08 (*QYrww.wgp.4A-3* and *QYrww.wgp.6B*) to 0.15 (*QYrww.wgp.2B-1*).

### Detection of Previously Reported *Yr* Genes/QTL

The 31 markers for the 18 previously reported *Yr* genes or QTL were successfully tested in the 857 winter wheat accessions and/or their reference genotypes. The presence and absence of the markers in the winter wheat panel are provided in **Table S3**. *Yr46* was not detected in any of the 857 accessions. The *Yr15* markers were present in four accessions (Mckay, Esperia, IDN09-15702A, and WB1529), but these entries were susceptible to some or all *Pst* races tested in the seedling stage, indicating no *Yr15* because *Yr15* is effective to the six tested races and all other races identified so far in the U.S. Of the 16 genes/QTL detected in the panel, *Yr5* was detected only in accession *Triticum spelta* Album, the original donor of the gene, and the remaining 15 genes or QTL were detected in various numbers of accessions, with the frequencies presented below.

### Frequencies of Stripe Rust Genes/QTL in the Winter Wheat Panel and Various Nurseries

After correction based on the phenotypic data, accessions considered having the resistance genes/QTL are listed in **Table S5**. The frequencies of the 51 genes or QTL in the winter wheat panel and various nurseries are presented in **Table 4**.

**Table 4:** Frequencies (%) of stripe rust resistance genes or quantitative trait loci (QTL) identified with reported markers or through genotyping by multiplexed sequencing genome-wide association study (GMS-GWAS) in the winter wheat panel and nurseries.

Gene/QTL	Frequencies (%) of genes or QTL in nurseries									
	Panel (857)	1711_WCDN (165)	1709_WWCMN (204)	1701_WRDN (185)	1702_WEDN (92)	1726_WURN (16)	1712_WHWN (86)	1715_WEWN (36)	1716_WSWN (30)	1718_WESR (43)
**Genes/QTL identified by markers**										
*Yr5*	0.12	0.61	0.00	0.00	0.00	0.00	0.00	0.00	0.00	0.00
*Yr9*	8.05	4.85	5.88	10.27	10.87	0.00	15.12	19.44	0.00	0.00
*Yr10*	22.75	31.52	20.59	31.35	0.00	25.00	18.60	16.67	30.00	18.60
*Yr15*	0.00	0.00	0.00	0.00	0.00	0.00	0.00	0.00	0.00	0.00
*Yr17*	29.05	39.39	23.53	15.14	34.78	12.50	60.47	36.11	0.00	20.93
*Yr18*	10.62	7.88	8.82	7.03	0.00	81.25	0.00	5.56	46.67	41.86
*Yr27*	2.22	3.03	0.98	4.32	1.09	0.00	0.00	0.00	6.67	2.33
*Yr30*	4.90	4.24	15.69	0.54	0.00	0.00	2.33	0.00	0.00	0.00
*Yr46*	0.00	0.00	0.00	0.00	0.00	0.00	0.00	0.00	0.00	0.00
*YrSP*	2.92	3.64	1.47	2.70	3.26	0.00	8.14	2.78	0.00	0.00
*YrTr1*	1.75	1.82	0.00	3.78	1.09	12.50	2.33	0.00	0.00	0.00
*Yr76*	1.75	1.82	0.98	3.78	3.26	0.00	0.00	0.00	0.00	0.00
*Yr78*	9.33	4.85	2.94	20.00	29.35	0.00	2.33	0.00	0.00	0.00
*QYrMa.wgp-1AS*	17.85	12.73	13.73	43.78	1.09	25.00	12.79	5.56	0.00	11.63
*QYr.wpg-1B.1*	17.85	12.73	14.71	32.97	13.04	31.25	15.12	13.89	6.67	9.30
*QYrel.wgp-2BS*	38.86	66.06	16.18	51.35	3.26	100.00	48.84	33.33	26.67	34.88
*QYrsk.wgp-3BS*	34.42	18.18	42.16	53.51	2.17	56.25	43.02	22.22	26.67	37.21
*QYrsk.wgp-4BL*	7.23	15.15	3.92	3.78	7.61	12.50	10.47	0.00	13.33	0.00
**Genes/QTL identified by GMS-GWAS**										
*Qyrww.wgp.1A-1*	11.09	6.06	5.88	18.38	0.00	50.00	18.60	0.00	20.00	20.93
*Qyrww.wgp.1A-2*	32.32	11.52	39.71	35.68	39.13	56.25	32.56	30.56	30.00	41.86
*Qyrww.wgp.1A-3*	6.42	4.24	2.45	13.51	0.00	31.25	8.14	8.33	10.00	0.00
*Qyrww.wgp.1B*	15.17	4.24	3.43	53.51	6.52	31.25	3.49	0.00	6.67	2.33
*Qyrww.wgp.1D-1*	9.45	7.88	12.25	14.05	0.00	6.25	4.65	13.89	20.00	2.33
*Qyrww.wgp.1D-2*	55.78	52.73	42.65	41.08	51.09	93.75	100.00	100.00	90.00	39.53
*Qyrww.wgp.1D-3*	6.65	6.06	3.43	3.78	3.26	12.50	15.12	19.44	13.33	9.30
*Qyrww.wgp.2A-1*	23.92	4.85	20.59	20.54	25.00	31.25	56.98	30.56	56.67	27.91
*Qyrww.wgp.2A-2*	6.77	7.88	2.94	10.27	5.43	18.75	6.98	5.56	3.33	6.98
*Qyrww.wgp.2B-1*	53.44	55.15	65.69	26.49	41.30	37.50	66.28	66.67	73.33	86.05
*Qyrww.wgp.2B-2*	12.14	19.39	15.69	9.73	0.00	6.25	12.79	8.33	13.33	6.98
*Qyrww.wgp.2B-3*	14.12	0.00	44.12	14.05	1.09	0.00	2.33	5.56	0.00	0.00
*Qyrww.wgp.2B-4*	6.10	3.40	3.40	6.50	1.20	0.00	1.20	8.30	23.3	4.70
*Qyrww.wgp.2D-1*	26.60	9.09	33.82	26.49	39.13	0.00	50.00	41.67	3.33	0.00
*Qyrww.wgp.2D-2*	27.54	8.48	24.02	24.86	39.13	50.00	50.00	69.44	0.00	34.88
*Qyrww.wgp.2D-3*	10.62	9.70	3.43	6.49	6.52	43.75	11.63	0.00	60.00	34.88
*Qyrww.wgp.2D-4*	4.43	0.00	0.00	2.70	0.00	12.50	8.14	44.44	0.00	18.60
*Qyrww.wgp.3A*	5.37	3.03	0.00	4.32	10.87	0.00	22.09	11.11	0.00	0.00
*Qyrww.wgp.3B*	3.38	0.00	0.00	4.32	0.00	43.75	0.00	8.33	16.67	13.95
*Qyrww.wgp.3D*	26.60	12.12	10.78	81.08	26.09	0.00	5.81	19.44	0.00	0.00
*Qyrww.wgp.4A-1*	7.00	6.06	4.90	3.78	0.00	37.50	11.63	22.22	0.00	20.93
*Qyrww.wgp.4A-2*	13.19	4.85	10.78	27.57	20.65	6.25	6.98	0.00	10.00	6.98
*Qyrww.wgp.4A-3*	21.47	29.70	25.00	25.41	27.17	18.75	10.47	0.00	0.00	0.00
*Qyrww.wgp.4B*	6.77	3.64	5.88	5.41	3.26	25.00	12.79	0.00	16.67	16.28
*Qyrww.wgp.5A*	6.88	5.45	8.33	2.16	9.78	12.50	4.65	8.33	10.00	18.60
*Qyrww.wgp.5B*	9.92	9.09	2.45	5.95	19.57	50.00	11.63	11.11	20.00	18.60
*Qyrww.wgp.5D*	7.58	3.64	4.41	1.62	13.04	43.75	17.44	11.11	10.00	13.95
*Qyrww.wgp.6A-1*	41.07	63.03	42.65	40.54	38.04	25.00	32.56	13.89	23.33	16.28
*Qyrww.wgp.6A-2*	48.07	59.39	49.02	51.35	53.26	50.00	32.56	44.44	30.00	20.93
*Qyrww.wgp.6B*	7.70	7.27	0.49	2.16	14.13	43.75	0.00	0.00	33.33	44.19
*Qyrww.wgp.7A-1*	5.37	3.64	2.45	1.62	9.78	43.75	2.33	22.22	3.33	11.63
*Qyrww.wgp.7A-2*	7.35	14.55	7.35	3.24	4.35	0.00	12.79	0.00	10.00	0.00
*Qyrww.wgp.7B-1*	7.00	0.00	0.00	15.68	3.26	12.50	12.79	19.44	16.67	6.98
*Qyrww.wgp.7B-2*	13.30	13.94	6.86	24.32	26.09	0.00	9.30	0.00	0.00	0.00
*Qyrww.wgp.7B-3*	13.30	19.39	4.90	3.78	13.04	68.75	27.91	22.22	16.67	11.63
Mean freq. (%)	14.76	13.24	12.86	17.29	12.47	24.88	17.42	15.72	14.65	13.87
No. of genes/QTL	51	45	43	48	37	35	43	33	32	31
Rate of gene/entry	0.06	0.27	0.21	0.56	0.40	2.10	0.50	0.91	1.07	0.72

Among the previously reported genes or QTL identified by markers, *Yr5*, *Yr9*, *Yr27*, *Yr30*, *YrSP*, *Yr76*, *YrTr1*, *Yr76*, *Yr78*, and *QYrsk.wgp-4BL* had low frequencies (< 10%); while *Yr10*, *Yr17*, *Yr18*, *QYrMa.wgp-1AS*, *QYr.wpg-1B.1*, *QYrel.wgp-2BS*, and *QYrsk.wgp-3BS* had relatively high frequencies (10.62 to 38.86%) in the panel (**Table 4**). These genes were not evenly distributed in the different nurseries. For instance, *Yr17* had a relatively high frequency in the Great Plains hard red nursery (1712_WHWN, 60.47%) compared to other nurseries (0%–39.39%). *Yr18*, *QYrel.wgp-2BS*, and *QYrsk.wgp-3BS* had relatively high frequencies in the western uniform regional nursery (1726_WURN, 56.25%–100%) compared to other nurseries.

Genes or QTL identified by GMS-GWAS also had a wide range of frequencies from 3.38% (*Qyrww.wgp.3B*) to 55.78% (*Qyrww.wgp.1D-2*) in the panel (**Table 4**). *Qyrww.wgp.1B* was highly present (53.51%) in the western regional nursery (1701_WRDN, 53.51%) that consisted of historical and recent cultivars grown in the U.S. Pacific Northwest (PNW) region, but was low in other nurseries, especially low in those from the eastern U.S. (1712_WHWN, 1715_WEWN, 1716_WSWN, and 1718_WESR). In contrast, *Qyrww.wgp.2A-1* and *Qyrww.wgp.2B-1* had relatively high frequencies in the eastern regional nurseries compared to the western ones.

### Effects of the Number of Resistance-Favorable Alleles on Stripe Rust Response

**Figure 6:**
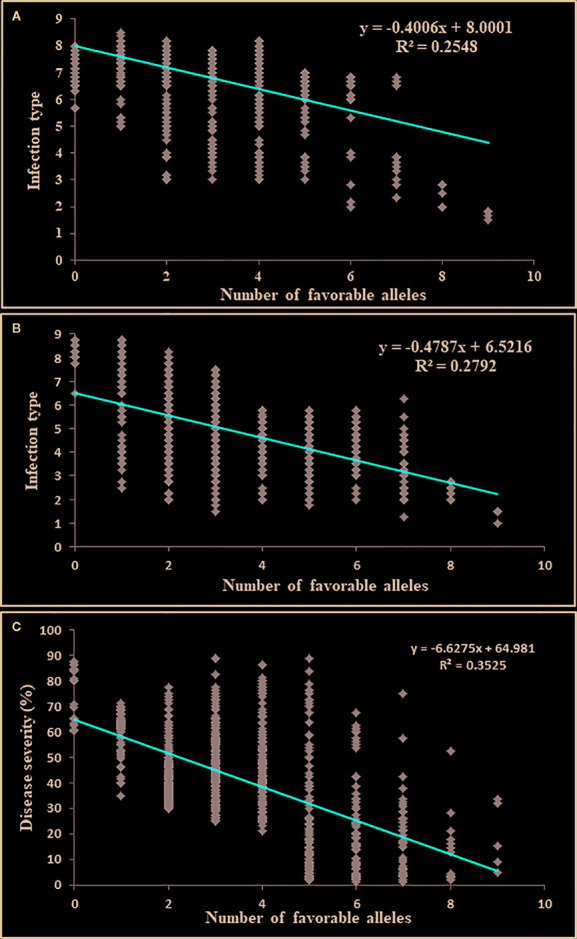
The effect of pyramiding multiple resistance alleles of stripe rust resistance genes or QTL identified in this study. Scatter plots of the number of resistance alleles versus IT data averaged across the seedling tests **(A)**, IT data averaged across the field tests **(B)**, and DS data averaged across the field tests **(C)**.

The markers representing the 35 QTL identified through GMS-GWAS were used to determine the number of resistance QTL in each of the 857 accessions. The number of resistance-favorable alleles in a single accession ranged between 0 and 9. The additive effect of resistance alleles was significant for both ASR and field resistance. The number of resistance alleles was negatively correlated with the IT and DS scores, with correlation coefficients −0.40 for ASR, and −0.47 and −0.62 for IT and DS, respectively in the field tests (*P* value ≤0.0001) (**Figure 6**). The *R* ^2^ values of the regression for ASR were 0.25, and 0.28 and 0.35 for field IT and DS of the field tests, respectively. The accessions that had relatively high numbers of the favorable alleles showed a comparatively low IT and DS.

## Discussion

In this study, we studied stripe rust resistance in 857 winter wheat accessions through testing with markers for 18 previous reported genes or QTL and with SNP markers generated through the GMS-GWAS approach. Based on the tests with previously reported markers, 16 previously known resistance genes were detected in at least one accession of the winter wheat panel, most of which were still valuable when combing with other *Yr* genes or QTL. Using GMS-GWAS, we identified 35 significantly associated stripe rust resistance loci including 15 loci associated with ASR and 20 associated with APR. All of the 51 genes or QTL were detected in various frequencies among the different nurseries, suggesting different intensities of use in breeding programs in different regions. This is one of the GWAS studies that use a large number of accessions and identify a large number of genes or QTL for an important trait of wheat. This study demonstrated that the GMS technique, which costs much less than the 9K or 90K SNP array, is efficient for mapping wheat genes when used in combination with the GWAS approach. More importantly, the study identified at least 10 potentially new genes and provided useful information about which genes in individual cultivars and breeding lines developed in various winter wheat growing regions in the United States.

Using molecular markers, we tested 18 previously reported genes/loci for stripe rust resistance in the winter wheat panel and successfully identified 16 of them in some of the accessions (**Table 4**). *Yr46*, which was reported in spring wheat for slow-rusting or APR to stripe rust (Herrera-Foessel et al., 2011), was not detected in any of the accessions. Similarly, this gene was also absent in the U.S. spring wheat panel (Liu et al., 2020). *Yr5* was detected only in its original donor *T. spelta* Album. The markers of *Yr15* were positive in four accessions. However, these accessions were susceptible to some of the tested *Pst* races. Because no race virulent to *Yr15* has been detected in the U.S. (Wan and Chen, 2014; Wan et al., 2016), we considered that these accessions do not have *Yr15*. Our results indicate that the *Yr15* markers may not be perfect, and further studies are needed to study the genomic regions covering the *Yr15* locus in these cultivars. The relatively high frequencies of *Yr10*, *Yr17*, *QYrMa.wgp-1AS*, *QYr.wpg-1B.1*, *QYrel.wgp-2BS*, and *QYrsk.wgp-3BS* indicate that these genes/QTL have been used quite extensively in breeding winter wheat cultivars in the U.S. Among these, *QYrMa.wgp-1AS, QYrel.wgp-2BS*, and *QYrsk.wgp-3BS* were identified in Madsen, Eltan, and Skiles, respectively, which have been the widely grown cultivars and used in breeding programs in the U.S. PNW (Liu et al., 2018; Liu et al., 2019a; Liu et al., 2019b). Of the 18 tested genes/QTL, 10 (*Yr5*, *Yr9*, *Yr10*, *Yr15*, *Yr17*, *Yr27*, *Yr76*, *YrSP*, *YrTr1*, and *QYr.wpg-1B.1*) confer ASR, while the other 8 (*Yr18*, *Yr30*, *Yr46*, *Yr78*, *QYrMa.wgp-1AS*, *QYrel.wgp-2BS*, *QYrsk.wgp-3BS*, and *QYrsk.wgp-4BL*) confer APR or HTAP resistance (Naruoka et al., 2015; Wang and Chen, 2017; Liu et al., 2018; Liu et al., 2019a; Liu et al., 2019b). Currently, for the ASR genes tested in the present study, only *Yr5* and *Yr15* are still resistant to all U.S. races of *Pst* identified thus far (Wan and Chen, 2014; Wan et al., 2016), and these genes have been successfully used in combination in spring wheat breeding as they provide complete protection (Liu et al., 2020). These genes together with *Yr46* and other effective genes detected in the present study should be used in breeding winter wheat cultivars.

In addition to the above previously known 16 genes/QTL, we detected 16 QTL with the greenhouse seedling data (**Table 2**) and 20 QTL using the adult-plant data from the fields (**Table 3**). We compared these genes/QTL to each other if they were mapped to the same chromosomal arms and also compared them with previously reported genes on the same chromosomal arms according to the marker positions, types and sources of resistance, and explained phenotypic variation.

On chromosome 1A, *QYrww.wgp.1A-1* and *QYrww.wgp.1A-2* were detected using the field adult-plant data and *QYrww.wgp.1A-3* with the greenhouse seedling data; and they all were mapped to 1AL but at positions 223, 518, and 571 Mb, respectively, indicating that they are different from each other. *QYrww.wgp.1A-1* was mapped to the similar region of *QYr.tam-1A* in U.S. variety TAM 111 (Basnet et al., 2014a). However, TAM 111 did not show the *QYrww.wgp.1A-1* marker in the present study. *QYrww.wgp.1A-2* was located in the same region as *QYr.sun-1A_Janz*, however, Janz is a spring wheat cultivar in Australia. Therefore, the relationship between *QYrww.wgp.1A-2* and *QYr.sun-1A_Janz* still needs a further study. *QYrww.wgp.1A-3* located at the long arm was identified with PSTv-4, and it was in the proximal region with *QYr.tam_1AL_TAM112* (Basnet et al., 2014a). However, TAM112 did not show the *QYrww.wpg.1A-3* marker. To determine the relationships of *QYrww.wgp.1A-1* with previously reported genes on 1AL from various wheat varieties (Wang and Chen, 2017) needs further studies. *QYrww.wgp.1B* was detected with all 6 races in the greenhouse seedling tests, but was not significant in the field tests, probably because many other loci contributed more significantly to the resistance in the fields. This QTL is different from *Yr9*, *Yr10*, *Yr15*, and *YrTr1* also on 1BS as it is effective against races different from those the other genes are resistant to. However, this QTL should be the same as *QYr.wpg-1B.1* reported in several winter wheat cultivars developed in the U.S. PNW (Naruoka et al., 2015), as they had the similar frequencies (15.17% and 17.85%) in the current panel.

Three QTL were detected on chromosome 1D, *QYrww.wgp.1D-1* and *QYrww.wgp.1D-2* on 1DS while *QYrww.wgp.1D-3* on 1DL. Although the two QTL on 1DS was only 4 Mb apart (**Table 3**), we treated them as different loci because relatively few markers on the D sub-genome. Moreover, *QYrww.wgp.1D-1* was detected with the IT and DS data in both Mount Vernon and Pullman in only 9.45% of the accessions, whereas *QYrww.wgp.1D-2* was detected in 55.78% of the accessions but only in Mount Vernon with the IT data. To date, six QTL for stripe rust resistance have been reported on chromosome 1D. All of them were in the short arm. *QYrww.wgp.1D-1* and *QYrww.wgp.1D-2* were mapped to the same regions of *QYrst.orr-1DS_Stephens* (Vazquez et al., 2012) and *QYr.caas-1DS_Naxos* (Ren et al., 2012), respectively. Stephens was found to have the resistance allele of *IWB2803* representing *QYrww.wgp.1D-1*. Thus, *QYrww.wgp.1D-1* could be the same as *QYrst.orr-1DS_Stephens*. Whether the resistance allele of marker *IWA1787* representing *QYrww.wgp.1D-2* is present in *QYr.caas-1DS_Naxos* is unknown. As *QYrww.wgp.1D-3* was mapped to 1DL and there are no previously reported genes/QTL for resistance to stripe rust on 1DL, this QTL should be new.

*QYrww.wgp.2A-1*, which conferred effective resistance detected at the adult-plant stage in all field environments, was mapped in the region of *Yr17* on chromosome 2AS. Wheat accessions, such as Jagger and Madsen, with *Yr17* had the resistance allele of *QYrww.wgp.2A-1* marker. As previously discussed, the *Yr17* marker region also contains a different gene for HTAP resistance (Liu et al., 2018; Liu et al., 2020). A study using ethyl methanesulfonate mutagenesis demonstrated that *Yr17* and the HTAP resistance genes are at different loci (Y. X. Li and X. M. Chen, unpublished data). Because the major races in the Mount Vernon and Pullman fields in 2018 and 2019 are virulent to *Yr17* (Liu et al., 2020), the resistance QTL effective in the fields should be the locus for HTAP resistance. *QYrww.wgp.2A-2* on chromosome 2AL was mapped in the region of *Yr1* (Wang and Chen, 2017) and *QYr.inra_2AL.2_Campremy* (Boukhatem et al., 2002). This QTL should be *Yr1* as both confer ASR.

Among the three QTL for the field resistance, *QYrww.wgp.2B-1* was mapped to the region of 2BS containing several genes/QTL for resistance to stripe rust (Wang and Chen, 2017). The region includes *Yr27* and *Yr31*. Two loci for HTAP resistances from U.S. PNW cultivar Luke were mapped to this chromosomal region (Guo et al., 2008). QTL in this region were also reported in PNW cultivars IDO444, Louise, and Stephens (Carter et al., 2009; Chen et al., 2012). However, Luke, IDO444, and Stephens did not have the resistance alleles of *QYrww.wgp.2B-1*, and spring wheat Louise was not in this panel. Thus, whether *QYrww.wgp.2B-1* is novel still needs an additional study. *QYrww.wgp.2B-2* and *QYrww.wgp.2B-3* were located in another region of chromosome 2BS without any previously reported *Yr* genes or QTL, and thus possibly new loci for stripe rust resistance. *QYrww.wgp.2B-4* conferring ASR to races PSTv-4 and PSTv-51 was located in the distal region of 2BL. To date, there was no any report of genes/QTL for stripe rust resistance in this region. Therefore, *QYrww.wgp.2B-4* is likely a new QTL.

Three QTL, including *QYrww.wgp.2D-1*, *QYrww.wgp.2D-2*, and *QYrww.wgp.2D-3*, for the resistance at the adult-plant stage in the fields were mapped to chromosome 2DS. The markers representing them have not been integrated into the consensus map. Thus, their novelty was still unknown. *QYrww.wgp.2D-4* conferring ASR to race PSTv-4 was located on 2DL. This gene should be different from previously reported APR genes *Yr54* and *Yr55* on 2DL (Basnet et al., 2014b; Wang and Chen, 2017), as they confer different types of stripe rust resistance. *Yr37* conferring ASR was originally from *Aegilops kotschyi* and located on 2DL (Marais et al., 2005). Although *QYrww.wgp.2D-4* is likely different from *Yr37* because of the different origin, their genetic distance needs to be determined.

Only one QTL each was mapped to chromosomes 3A, 3B, and 3D. The three QTL were detected only with one race or in one field environment. The position region of *QYrww.wgp.3A* represented by *IWB44443* is different from previously reported QTL on 3AL (Wang and Chen, 2017), and therefore, it is likely a new QTL. The relationships of *QYrww.wgp.3B* and *QYrww.wgp.3D* with previously identified QTL on 3BL or 3DL (Wang and Chen, 2017) require further studies.

Three QTL were mapped on chromosome 4A and one QTL on 4B. *QYrww.wgp.4A-1* on 4AL was different from previously identified QTL, whereas *QYrww.wgp.4A-2,* also on 4AL, was in the region of *Yr51* (Randhawa et al., 2013). Thus, *QYrww.wgp.4A-2* and *Yr51* are possibly the same gene or tightly linked genes. *QYrww.wgp.4A-3* mapped to 4AL and *QYrww.wgp.4B* to 4BL each were detected with only one environment data. Their relationships with previously mapped genes on these chromosome arms are not clear.

Three QTL for ASR were mapped on the group 5 chromosomes, with *QYrww.wgp.5A* on 5AL, *QYrww.wgp.5B* on 5BL, and *QYrww.wgp.5D* on 5DS. Based on their marker positions (**Tables 2** and **3**), *QYrww.wgp.5A* and *QYrww.wgp.5B* are different from other QTL previously reported on the same chromosomal arms (Wang and Chen, 2017). *QYrww.wgp.5D* was mapped in the distal end of 5DS. To date, two QTL for resistance to stripe rust have been reported on 5DS. Ren et al. (2015) mapped *QYr.caas-5DS* in Chinese cultivar “Jingshuang 16” near the centromere of 5D, and the QTL explained 5.1%–18.0% phenotypic variance at the adult-plant stage across different environments. Singh et al. (2000) reported *QYr-5DS* for APR to stripe rust in Mexican cultivar Opata 85 in the same region according to the consensus SSR map (Somers et al., 2004). As our 5DS QTL confers ASR, it is likely a different gene.

Two QTL were identified on chromosome 6A. *QYrww.wgp.6A-1* conferring ASR was mapped on 6AL. *QYrtb.orz-6AL*, a major QTL for stripe rust resistance in U.S. PNW winter wheat Tubbs, was previously mapped to this region (Vazquez et al., 2015). Tubbs was found to have the resistance allele of *IWB52712* representing *QYrww.wgp.6A-1*, and both QTL have the same resistance type and resistance source. Therefore, *QYrww.wgp.6A-1* and *QYrtb.orz-6AL* are likely the same QTL. *QYrww.wgp.6A-2* was located in a different region from previously mapped QTL on 6AL, and thus, it is likely a new QTL. *QYrww.wgp.6B* was mapped to 6BL. This QTL is likely the same as *QYrdr.wgp-6BL.1* identified from Druchamp by Hou et al. (2015), as this European variety has been used in U.S. PNW wheat breeding programs (Chen, 2013).

*QYrww.wgp.7A-1* was located at the same region as *QYr.caas-7A* on 7AS in Chinese cultivar Jingshuang 16 (Ren et al., 2015). However, the sources of these QTL are quite different. Thus, they may be different genes, but a clear conclusion is needed from additional studies. Three QTL were identified on chromosome 7B, all on the long arm. *QYrww.wgp.7B-1* identified from the field experiments was close to *QYr.sun-7B*, which was identified in Australian cultivar Kukri (Bariana et al., 2010). These two QTL are likely the same as both had moderate LOD and PEV values. *QYrww.wgp.7B-2* was mapped to the same location as *QYr.caas-7B.1* (Ren et al., 2012), and these QTL could be the same. *QYrww.wgp.7B-3* was mapped to a different region with previously mapped genes or QTL, and thus, should be a novel gene for resistance to stripe rust.

## Conclusions

In summary, using the combination of testing previously known genes and GMS-GWAS, we detected a total of 51 loci for stripe rust resistance in the panel of 857 winter wheat accessions including genetic stocks, breeding lines and cultivars used and developed in the U.S. These genes or QTL confer either race-specific ASR or race nonspecific APR. At least ten of the QTL (*QYrww.wgp.1D-3*, *QYrww.wgp.2B-2*, *QYrww.wgp.2B-3*, *QYrww.wgp.2B-4*, *QYrww.wgp.3A*, *QYrww.wgp.5A*, *QYrww.wgp.5B*, *QYrww.wgp.5D*, *QYrww.wgp.6A-2* and *QYrww.wgp.7B-3*) are likely novel, which enhance the diversity of stripe rust resistance. Both *Yr5* and *Yr15*, which are effective against all *Pst* races identified thus far in the U.S., were not found in any breeding lines or commercially grown cultivars. The present study provides the information about which genes in the breeding lines and commercially grown cultivars in various regions of winter wheat production in the U.S., which is important for managing stripe rust by deploying the currently available resistant cultivars and utilizing the resources of resistant genotypes, genes and markers for breeding new cultivars with resistance to stripe rust. The genes identified in the current study should be used, together with many other genes reported in the literature (Wang and Chen, 2017; Liu et al., 2020), in wheat breeding programs to enhance the diversity, overall level and durability of stripe rust resistance. More studies should be conducted to dissect the chromosome regions containing several potentially the same or tightly linked genes for resistance to stripe rust and other diseases, and to convert the SNP markers into user-friendly KASP markers for more efficiently incorporation of genes for effective resistance into new cultivars.

## Author’s Note

Mention of trade names or commercial products in this publication is solely for thepurpose of providing specific information and does not imply recommendation orendorsement by the U. S. Department of Agriculture. USDA is an equal opportunityprovider and employer.

## Data Availability Statement

The datasets generated for this study can be found in Figshare https://doi.org/10.6084/m9.figshare.12409181.v1.

## Author Contributions

JM and LL conducted experiments, analyzed and interpreted data, and draft and revised the manuscript. YL and MW participated in the experiments. DS provided resources and technique guidance. DH provided resources and guidance. XC developed and guided the project, interpreted data, and wrote the manuscript. All authors contributed to the article and approved the submitted version.

## Conflict of Interest

The authors declare that the research was conducted in the absence of any commercial or financial relationships that could be construed as a potential conflict of interest.
