# Supplementary material for: Genome-Wide Association Study and Gene Specific Markers Identified 51 Genes or QTL for Resistance to Stripe Rust in U.S. Winter Wheat Cultivars and Breeding Lines
Source: Front Plant Sci. 2020 Jul 3;11:998. doi: 10.3389/fpls.2020.00998 (PMC7350909; doi:10.3389/fpls.2020.00998)
Supplement: Supplementary file 1 [file Table_1.docx]

**Supplementary Table S1.** Virulence formulae and octal codes of five *Puccinia striiformis* f. sp. *tritici* races used in the seedling tests

| Race | Octal code | **Virulence**/avirulence formula^a^ |
| --- | --- | --- |
| PSTv-4 | 511211 | **1,6,9,17,27,SP,76**/5,7,8,10,15,24,32,43,44,Tr1,Exp2 |
| PSTv-14 | 571267 | **1,6,7,8,9,17,27,43,44,Tr1,Exp2,76**/5,10,15,24,32,SP |
| PSTv-37 | 171266 | **6,7,8,9,17,27,43,44,Tr1,Exp2**/1,5,10,15,24,32,SP,76 |
| PSTv-40 | 174766 | **6,7,8,9,10,24,27,32,43,44,Tr1,Exp2**/1,5,15,17,SP,76 |
| PSTv-51 | 575777 | **1,6,7,8,9,10,17,24,27,32,43,44,SP,Tr1,Exp2,76**/5,15 |
| PSTv-198 | 170262 | **6,7,8,9,27,43,44,Exp2**/1,5,10,15,17,24,32,SP,Tr1,76 |

^a^ The octal codes and virulence formula were based on the 18 *Yr* single-gene differentials: *Yr1*, *Yr5*, *Yr6*, *Yr7*, *Yr8*, *Yr9*, *Yr10*, *Yr15*, *Yr17*, *Yr24*, *Yr27*, *Yr32*, *Yr43*, *Yr44*, *YrSP*, *YrTr1*, *YrExp2*, and *Yr76* (Wan and Chen, 2014; Wan et al., 2016).
